# Diagnostic, prognostic and predictive value of cell-free miRNAs in prostate cancer: a systematic review

**DOI:** 10.1186/s12943-016-0523-5

**Published:** 2016-05-18

**Authors:** Edgars Endzeliņš, Vita Melne, Zane Kalniņa, Vilnis Lietuvietis, Una Riekstiņa, Alicia Llorente, Aija Linē

**Affiliations:** Latvian Biomedical Research and Study Centre, Ratsupites Str 1, k-1, LV-1067 Riga, Latvia; Riga Stradiņš University, Dzirciema Str 16, Riga, LV-1007 Latvia; Faculty of Medicine, University of Latvia, 19 Raina blvd., Riga, LV-1586 Latvia; Department of Molecular Cell Biology, Institute for Cancer Research, Oslo University Hospital-The Norwegian Radium Hospital, 0379 Oslo, Norway

**Keywords:** Prostate cancer, Cell-free miRNAs, Extracellular vesicles, Exosomes, Microvesicles, Biomarkers, Liquid biopsy, Overdiagnosis

## Abstract

Prostate cancer, the second most frequently diagnosed cancer in males worldwide, is estimated to be diagnosed in 1.1 million men per year. Introduction of PSA testing substantially improved early detection of prostate cancer, however it also led to overdiagnosis and subsequent overtreatment of patients with an indolent disease. Treatment outcome and management of prostate cancer could be improved by the development of non-invasive biomarker assays that aid in increasing the sensitivity and specificity of prostate cancer screening, help to distinguish aggressive from indolent disease and guide therapeutic decisions. Prostate cancer cells release miRNAs into the bloodstream, where they exist incorporated into ribonucleoprotein complexes or extracellular vesicles. Later, cell-free miRNAs have been found in various other biofluids. The initial RNA sequencing studies suggested that most of the circulating cell-free miRNAs in healthy individuals are derived from blood cells, while specific disease-associated miRNA signatures may appear in the circulation of patients affected with various diseases, including cancer. This raised a hope that cell-free miRNAs may serve as non-invasive biomarkers for prostate cancer. Indeed, a number of cell-free miRNAs that potentially may serve as diagnostic, prognostic or predictive biomarkers have been discovered in blood or other biofluids of prostate cancer patients and need to be validated in appropriately designed longitudinal studies and clinical trials. In this review, we systematically summarise studies investigating cell-free miRNAs in biofluids of prostate cancer patients and discuss the utility of the identified biomarkers in various clinical scenarios. Furthermore, we discuss the possible mechanisms of miRNA release into biofluids and outline the biological questions and technical challenges that have arisen from these studies.

## Background

Prostate cancer is a global health problem. Approximately 1.1 million cases are diagnosed per year, making this malignancy the second most common cancer in men worldwide and the most common cancer in men in more developed regions [1, 2]. In terms of mortality, prostate cancer is the fifth leading cause of death from cancer in men [1, 2].

In the economically developed countries, over 80 % of prostate cancer cases are diagnosed at localised stage [3], when the disease can often be cured by localised therapies such as radical prostatectomy and radiotherapy. Technical developments in radical prostatectomy as well as targeted external beam radiation therapy have significantly reduced patient morbidity after curative treatment. Cancer specific survival 5 years after the time of diagnosis is high for localised prostate cancer, and it reaches almost 100 % in USA according to the American Cancer Society. However, the more advanced the cancer at diagnosis, the poorer the prognosis. When metastatic prostate cancer is diagnosed, androgen deprivation is the initial line of therapy. Androgen deprivation therapy (ADT), however, is a palliative and not a curative treatment for patients with metastases, and eventually the patients will develop metastatic castration-resistant prostate cancer (mCRPC), for which currently available treatment options have limited efficacy [4, 5]. Once the disease is androgen independent, the estimated 5-year survival drops to 28 % and the average survival time is 2 years [4].

The discovery of prostate specific antigen (PSA) almost 30 years ago has changed the way how prostate cancer is diagnosed and managed. The serum PSA test is currently the most commonly used tool for organised screening programs, opportunistic screening and monitoring of prostate cancer. Evidence obtained in numerous clinical trials suggests that the PSA test may improve the early detection of localised prostate cancer, however it has substantial drawbacks due to overdiagnosis and overtreatment. The balance of benefits and harms is still a matter of active debate, and improving the performance of PSA-based screening for prostate cancer is essential [6–8]. Furthermore, recent advances in the development of therapeutics for prostate cancer have raised the necessity for biomarkers that can predict treatment outcome and be used in therapeutic decisions. It is clear that there is clinical need for novel prostate cancer biomarkers. The identification of cancer biomarkers that can be measured in a noninvasive way, for example in a blood or urine sample, is of particular importance as these samples can be easily acquired throughout the course of the disease. These biomarkers, often referred as circulating biomarkers or liquid biopsies [9], may better reflect the heterogeneity of the tumour than single biopsies.

In 2008, three independent studies demonstrated that tumour-associated miRNAs are released into the blood circulation and are present in human plasma and serum in a remarkably stable form [10–12]. More recently, cell-free miRNAs have also been found in a variety of other biofluids [13–15]. Given that miRNA expression patterns are tissue and cancer-type specific [16, 17], these findings led to the concept that different cancers may leave specific miRNA signatures in biofluids [12], and that these signatures may carry information about the disease status, aggressiveness and response to therapy. This concept has attracted enormous attention of researchers resulting in the discovery of cell-free miRNA signatures with diagnostic, prognostic and predictive relevance for various types of cancer, including prostate cancer. In the current review, we systematically summarise studies exploring cell-free miRNAs in biofluids of prostate cancer patients, propose their clinical utility in various clinical scenarios and discuss mechanisms for miRNA release in biofluids.

### Unmet clinical needs in the management of prostate cancer

Prostate cancer is a multi-faceted disease and clinicians treating and managing the disease face several challenges at the different clinical states [18]. The first decision point is the early detection of localised tumours. Since prostate cancer symptoms generally appear at advanced stages of the disease, PSA-based screening seemed an appealing idea and many countries launched population-based screening programs in the early 1990s [6–8]. A combination of high PSA levels in blood and a positive digital rectal exam typically leads to a biopsy to confirm diagnosis and determine the Gleason grade. PSA-based screening indeed has proved useful in detecting early stage prostate cancer and has been shown to reduce the rate of death from prostate cancer in some studies [19]. However, PSA is not cancer specific – it is a glycoprotein produced by normal prostate epithelial cells at equal or higher levels than by cancer cells and released into the bloodstream due to increased epithelial barrier permeability and cellular reorganisation [20]. Elevated serum PSA levels can be found not only in men with prostate cancer, but also in men with benign prostatic hyperplasia (BPH) and prostatitis [21–23]. Moreover, a variety of factors such as ejaculation, prostate biopsy, acute urinary retention and even bicycle riding may transiently increase PSA levels [24, 25]. In fact, several initial studies have demonstrated that only 22–26 % of men with elevated PSA levels (4.0–9.9 ng/ml) have cancer [26–28]. High false positive rate and low specificity of the PSA test consequently leads to large numbers of unnecessary prostate biopsies and emotional morbidity [8, 29–31]. The PSA-based test also gives a high rate of false negatives. For instance, a study by Thompson et al. involving 2950 men with PSA levels ≤4.0 ng/ml showed that 15.2 % of them had a biopsy-detected prostate cancer [32]. Several alternative approaches for improving the diagnostic performance of PSA have been suggested, including PSA velocity, PSA density, age-specific PSA levels and free to total PSA ratio [33]. However all these tests have their own advantages and drawbacks, and their clinical utility has not been validated in randomised trials so far. Hence, additional or alternative biomarkers that could increase the sensitivity and specificity of prostate cancer screening still represent an unmet clinical need.

The data about the impact of PSA-based screening on prostate cancer mortality are controversial. While the European Randomized Study of Screening for Prostate Cancer that was initiated in the early 1990s and involved 182,000 men concluded that PSA-based screening reduced the death rate by 20 % [6, 19], the Prostate, Lung, Colorectal, and Ovarian (PLCO) Cancer Screening Trial that was carried out in the USA between 1993 and 2001 and enrolled 76,685 men found no evidence of reduced mortality [7]. The reason for such a discrepancy is not yet clear. Both studies, however, concluded that organised PSA-based screening is associated with high risk of overdiagnosis. In line with this conclusion, a recent Norwegian study focusing on the effects of opportunistic PSA testing on trends in stage distribution in different age groups observed a considerable increase in the incidence of localised prostate cancer in younger men, and a moderate decrease in the incidence of advanced cancer in older men, but that did not fully compensate the initial increase [29]. Overdiagnosis refers to the detection of indolent cancers that most likely would never have become clinically significant and leads to aggressive treatments with substantial side effects [29, 34]. The overdiagnosis rate for annual PSA-based screening has been estimated to be approximately 50 %, and it is considered to be the most significant harm of screening [29, 34]. Once early stage prostate cancer is detected, the second decision point is to choice between radical treatment and active surveillance. Management decisions are based on risk stratification systems. For example, the D’Amico risk system stratifies the patients in low-, intermediate- and high-risk groups for recurrence after curative treatment based on PSA levels, Gleason score, and tumour stage [35]. However, actual risk classification systems (D’Amico risk-group classification, National Comprehensive Cancer Network, CAPRA score) often give different results and are not sufficient to distinguish true indolent from clinically significant prostate cancer thus resulting in many patients with indolent cancers being treated. There is a clear unmet need for biomarkers that can reduce overtreatment.

The third decision point is the choice of treatment for metastatic prostate cancer. The standard treatment for metastatic hormone-sensitive prostate cancer is ADT. Although most of the patients initially respond well, the disease ultimately becomes resistant and recurs within 1–3 years as mCRPC [4, 36]. Three recent clinical trials evaluated the effect of the addition of various chemotherapeutic agents to ADT – two of these studies, STAMPEDE [37] and CHAARTED [38], showed significant survival benefit for patients receiving ADT in combination with docetaxel, while the third study, GETUG-AFU 15, observed a trend towards improved survival, however it did not reach a statistical significance [39]. For mCRPC, the first-line therapy currently is docetaxel-based chemotherapy, which provides modest survival benefits (approximately 2.4 months compared with mitoxantrone-based therapy) and improve the quality of life in approximately 22 % of patients [40]. In the past five years, several novel chemotherapeutic (cabazitaxel) and radiopharmaceutical (radium-223) agents, androgen receptor-targeted agents (abiraterone acetate, and enzalutamide) and an immunotherapeutic approach (sipuleucel-T) have been approved for the management of mCRPC, all of them have been shown to improve overall survival by 3–5 months [41, 42]. More recently, several novel therapeutic agents targeting DNA-repair defects (Olaparib) [43], bone microenvironment and metastasis formation (Tasquinimod) [44], resistance pathways (Custirsen) [45] and mesenchymal-epithelial transition (Cabozantinib) [46] have been shown to have antitumour activity and survival benefits in subgroups of patients [47]. Therefore, the identification of biomarkers that could predict patients’ response to specific therapies and could help to select the optimal combination of agents or treatment schedule is becoming increasingly important [48].

### miRNAs are found in biofluids

miRNAs are a class of endogenous 19–22 nucleotides long, single-stranded non-coding RNA molecules. These small RNAs post-transcriptionally regulate gene expression by base-pairing to the complementary sites in their target mRNAs resulting either in the degradation of the target mRNAs or in the inhibition of translation initiation [49, 50]. The current release of miRBase (June, 2014) contains 1881 precursors and 2588 mature human miRNA sequences [51]. miRNA expression profiles have been found to be tissue type-specific and frequently dysregulated in various cancers. Importantly, they may robustly distinguish tumours from normal tissues and classify tumours according to the developmental lineage, differentiation state and aggressiveness [16, 52]. In prostate cancer, miRNA signatures that distinguish malignant from normal tissues, low- from high-risk cancers and recurrent from non-recurrent cancers have been identified [53–56].

Mitchell et al. demonstrated for the first time that miRNAs from human prostate cancer xenografts were released into the circulation of xenograft-bearing mice and could be readily detectable in the plasma once the tumours were established [11]. This study also showed that miRNAs in human plasma remain stable after incubation of plasma at room temperature for up to 24 h or multiple freeze-thaw cycles. Furthermore, serum levels of miR-141 were substantially higher in patients with metastatic prostate cancer than healthy controls and distinguished cases from controls with a sensitivity of 60 %, specificity of 100 % and an area under the curve (AUC) of 0.907 [11].

Cell-free miRNAs have been subsequently found in various human body fluids including blood, urine, tears, breast milk, bronchial lavage, colostrums, and also in seminal, amniotic, pleural, peritoneal and cerebrospinal fluid [13, 14, 57]. The highest number of detectable miRNAs was found in saliva, seminal fluid and breast milk, while urine, pleural fluid and cerebrospinal fluid had the lowest number of different miRNAs. The most abundant miRNAs were typically found in most of the biofluids, however there are miRNAs that were uniquely detected in a particular biofluid [13].

**Fig. 1:**
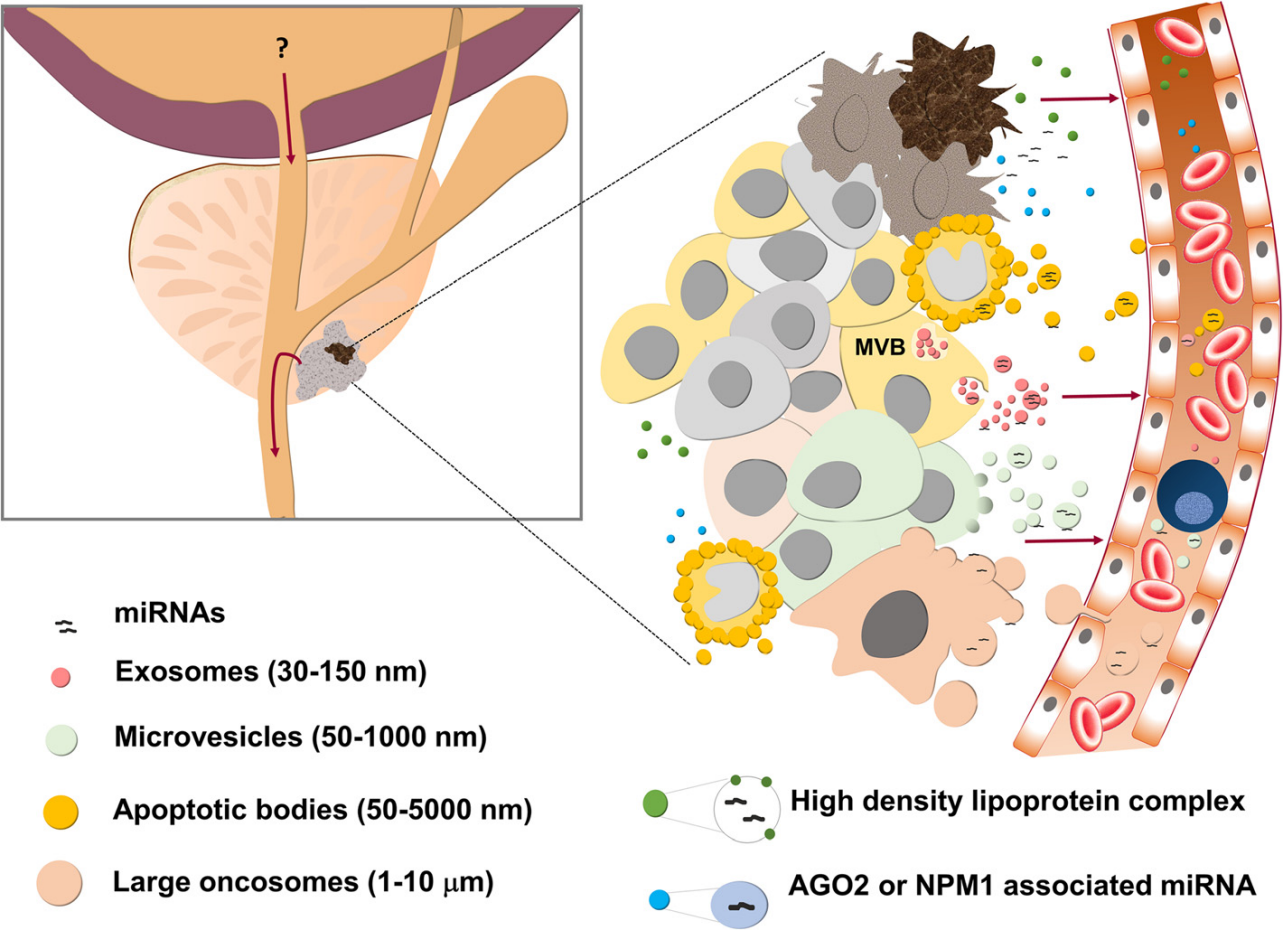
Different mechanisms of cell-free miRNA release from cancer cells. miRNAs can be released from cells and enter the bloodstream, urine or seminal fluid either in the form of membrane-enclosed vesicles (i.e., exosomes, microvesicles, apoptotic bodies and large oncosomes) that differ in size and molecular content or vesicle-free forms, where miRNAs are bound to specific RNA binding proteins or packaged in high density lipoprotein complexes. AGO2, argonaute2; NPM1, nucleophosmin 1; MVB, multivesicular body

In biofluids and cell culture media, cell-free miRNAs have been found to be packaged into extracellular vesicles (EVs) [58–62], or to exist in a vesicle-free form associated with proteins such as Argonaute2 [63, 64] and nucleophosmin [65], or high-density lipoproteins [66] (Fig. 1).

The proportion of vesicle-enclosed and vesicle-free miRNAs in biofluids is still a controversial issue. A study by Arroyo et al. demonstrated that only a minority of cell-free miRNAs in human plasma and serum are associated with EVs, while approximately 90 % of miRNAs are incorporated in Argonaute2 containing ribonucleoprotein complexes. Moreover, some miRNAs were exclusively associated with vesicles and others with ribonucleoprotein complexes [63]. The same group later performed a stoichiometric analysis of the miRNA content of exosomes isolated from various sources and found on average 0.00825 miRNA molecules per exosome. The authors proposed two models for exosomal RNA content: one suggests that a small fraction of exosomes carries a low concentration of miRNA and the other that very rare exosomes carry many copies of a given miRNA [67]. On the contrary, a study by Gallo et al. showed that the concentration of miRNAs was consistently higher in exosomal fractions as compared to exosome-depleted serum and saliva [68]. Cheng et al. performed deep sequencing of miRNAs in exosomal and total cell-free RNA fractions in human plasma and serum and found that exosomes are enriched in miRNAs and provide a consistent source of miRNAs for biomarker discovery [69]. Similarly, deep sequencing of exosomal and total cell-free small RNAs in human urine showed a significant enrichment of miRNAs in exosomes [69] and the level of exosomal, but not vesicle-free, miR-373 has been shown to be increased in patients with breast cancer [70]. It is important to note that all these studies are not directly comparable as they differ in the EV isolation methods, RNase and proteinase K treatment and possibly pre-analytical variables.

### EV-mediated miRNA release

The term “EV” refers to virtually any type of lipid bilayer-bound vesicle released into the extracellular space by any type of cell. EVs differ in their biogenesis, molecular content, size, membrane composition, cellular source and specific functions. According to the mode of biogenesis, three main types of EVs have been defined: (i) exosomes, (ii) microvesicles (MVs) and (iii) apoptotic bodies [71, 72] (Fig. 1). Exosomes are the smallest (30–150 nm) vesicles and formed via inward budding of endosomal membranes, resulting in multivesicular bodies (MVB) that later fuse with the plasma membrane releasing their internal vesicles/exosomes into the extracellular environment [73, 74]. Microvesicles are larger (50–1000 nm) vesicles generated by outward budding and fission of the plasma membrane [75, 76]. Apoptotic bodies are produced by dying cells during the late stages of apoptosis, when cells disassemble into membrane-bound vesicles ranging in size from 50 nm to 5 μm [73, 77]. Apoptotic bodies are promptly cleared by phagocytes in vivo and their main biological function is to facilitate the removal of aged or damaged cells and to prevent the leakage of their intracellular content in the extracellular space, thus minimising tissue damage and preventing the development of chronic inflammatory and autoimmune diseases [78]. More recently, atypically large EVs (1–10 μm), termed “large oncosomes”, were found to be generated by the shedding of non-apoptotic plasma membrane blebs from fast-migrating prostate cancer cells that had acquired an amoeboid phenotype known to be associated with aggressive or metastatic disease [79–81]. These vesicles were shown to harbour cancer-promoting bioactive molecules and to be more abundant in plasma of patients with metastatic prostate cancer in comparison to localised cancer, therefore they may be of particular importance as prognostic biomarkers [80, 82, 83]. It is not clear at the moment if large oncosomes represent a new type of EVs or a subtype of MVs.

EVs have been shown to contain a large variety of small non-coding RNA species, including miRNAs, tRNAs, snoRNAs, snRNAs, mitochondrial-associated RNA, piRNAs, vault RNAs and Y-RNAs, as well as mRNAs, lncRNAs and rRNAs [58, 59, 62, 84, 85]. Apparently, the EV RNA content is not merely a reflection of cellular RNA content, and some specific small RNAs are selectively exported to EVs, while others are excluded [85, 86]. Furthermore, the repertoire and proportions of various RNAs seems to differ between various types of EVs [80, 87, 88]. For example, in a recent study Lunavat et al. compared the small RNA content in exosomes, MVs and apoptotic bodies released by melanoma cells and found that exosomes were relatively enriched in small RNAs, while MVs and apoptotic bodies contained a larger proportion of rRNAs. Deep sequencing of miRNAs revealed a set of 113 miRNAs that were shared between all types of EVs and cells, a set of 23 miRNAs that were only detected in exosomes and a distinct set of 26 miRNAs that were shared between MVs, apoptotic bodies and cells, but not found in exosomes [87]. Hence, the sorting signals and mechanisms are likely to be different in distinct types of EVs. In this regard, several sorting mechanisms have been proposed. A study by Gibbings et al. demonstrated that GW182, a component of the RNA-inducing silencing complex, is localised in endosomal/MVB membranes and secreted in exosomes and thus it may be implicated in the loading of miRNAs into exosomes [89]. Later, Villarroya-Beltri et al. reported the identification of short sequence motifs in miRNAs that guide their sorting into exosomes and showed that sorting was mediated by sumoylated heterogeneous nuclear ribonucleoprotein A2B1 [90]. A study by Koppers-Lalic et al. suggested that, at least in B cells, sorting of miRNAs in exosomes depends on the 3′ end modifications – 3′-end uridylated miRNAs are preferentially sorted into exosomes, while 3′-end adenylated miRNAs are retained in cells [91]. At the same time, another showed that the loading of miRNAs into EVs is mediated by Annexin A2 in a sequence independent manner [92].

An important question is how EVs secreted by various cells reach the systemic circulation. After release from donor cells, EVs may be taken up by neighboring cells, internalized by the same donor cell or enter the systemic circulation to reach different tissues. One possible mechanism for EV entry into the blood stream could be by weakening the tight junctions in epithelial/endothelial barriers. Tight junctions are formed by protein complexes consisting of occludin and claudins, which are tetraspanins consisting of 4 transmembrane domains that form 2 extracellular loops and 1intracellular loop. The cytosolic C terminus of tetraspanins is linked to adaptor proteins ZO-1, ZO-2, ZO-3, which interact with the actin cytoskeleton ensuring the maintenance of tight junctions and epithelial barrier integrity [93]. In a recent study Zhou et al. demonstrated that cancer exosome-derived miR-105 can effectively weaken the tight junctions between endothelial cells by reducing ZO-1 expression in endothelial cells hence making the endothelial barrier more permeable for cancer cells [94]. Tominaga et al. have provided evidence that actin dynamics in the blood-brain barrier (BBB) is altered by miR-181c transferred by metastatic cancer-derived EVs [95]. miR-181c promotes the breakdown of BBB through the downregulation of its target gene, *PDPK1,* that results in abnormal localization of actin. Altogether, exosomal miRNAs from cancer cells have been shown to alter the epithelial/endothelial barrier permeability and may help EV entry into the systemic circulation.

### Cell-free miRNA signatures with diagnostic, prognostic or predictive relevance for prostate cancer

Following the initial discovery by Mitchell et al. [11] providing a proof of principle that miRNAs from prostate cancer cells are released in the bloodstream, where they are protected against degradation and readily detectable by PCR-based methods, a number of studies have explored miRNAs in biofluids of prostate cancer patients. The levels of specific miRNAs have been correlated with disease status, stage, aggressiveness and response to therapy. The main findings of these studies are summarised in Table 1.

**Table 1:** Studies investigating cell-free miRNA signatures with diagnostic, prognostic and predictive relevance in prostate cancer

Study design, substrate, and sample size	Significant miRNAs	Diagnostic value/outcome	Reference
TaqMan qRT-PCR analysis of 6 candidate miRNAs in serum from 25 mPC patients and 25 HI. Normalised to spike-ins.	**miR-141**	AUC = 0.907, Sn = 60 %, Sp = 100 %	Mitchell et al. 2008 [11]
miRNA profiling by in serum from 6 PC patients (stage 3 and 4) and 8 HI by pan-miRNA microarray.	**miR-16**, miR-34b, miR-92a, miR-92b, **miR-103**, **miR-107**, miR-197, miR-328, miR-485-3p, miR-486-5p, **miR-574-3p**, miR-636, miR-640, miR-766, miR-885-5p	Upregulated miRNA levels in PC serum samples	Lodes et al. 2009 [118]
qRT-PCR analysis of 3 candidate miRNAs in plasma from 51 PC patients (26 LPC, 25mPC) and 20 HI. Normalized to RNU1A.	**miR-21, miR-141, miR-221**	miR-21: AUC = 0.880 (PC vs HI)	Agaoglu et al. 2011 [119]
		miR-221: AUC = 0.830 (PC vs HI)	
		miR-141: AUC = 0.755 (mPC vs LPC)	
Discovery: profiling of 667 miRNAs in serum of 7 mPC and 14 LPC patients by TaqMan arrays. Normalised to spike-ins.	**miR-141, miR-200b**, **miR-375**	69 differentially expressed miRNAs between mPC and LPC.	Brase et al. 2011 [120]
Validation 1: analysis of 5 selected miRNA in different PC risk groups (n = 45).		Increased miR-375, miR-141, miR-200b levels differentiate pT3 vs pT2, Gleason score 6 vs 7.	
Validation 2: analysis of 3 selected miRNA in different PC risk groups (n = 71).		miR-375 level differentiate Gleason score 7 vs ≥8 and N0 vs N1/M+	
qRT-PCR analysis of 4 candidate miRNAs in serum from 45 PC, 18 BPH patients and 20 HI. Normalised to spike-ins.	let-7i, miR-26a, miR-32, miR-195	4 miRNA model: AUC = 0.758, Sn = 78,4 %, Sp = 66.7 % (LPC vs BPH) (PSA AUC = 0.834)	Mahn et al. 2011 [121]
		Decrease of miR-26a and miR-195 level after prostatectomy	
Profiling of 384 miRNAs in serum from 36 PC patients (CAPRA scores: 12 low, 12 medium, 12 high risk) and 12 HI using Fluidigm microfluidic platform, validation of 12 candidate miRNAs by qRT-PCR in the same sample set.	**miR-24**, **miR-26b**, **miR-30c, miR-93**, **miR-106a**, **miR-223**, **miR-451**, **miR-874**, miR-1207-5p, miR-1274a	10 differentially expressed miRNAs (PC vs HI)	Moltzhan et al. 2011 [100]
		miR-106a, miR-93, miR-1274: a liner relationship between increased miRNA level and increased risk score.	
		miR-24: a liner relationship between decreased miRNA level and increased risk score.	
		miR-451: increased in high risk in comparison to low and medium risk PC and HI.	
qRT-PCR analysis of miR-21 in serum from 56 patients (20 LPC, 20 ADPC, 10 CRPC and 6 BPH) The 10 CRPC patients received docetaxel-based chemotherapy.	**miR-21**	A liner relationship between increased miR-21 level and increased serum PSA level in patients with ADPC and HRPC.	Zhang et al. 2011 [122]
		Higher miR-21 levels in patients who were resistant to docetaxel-based chemotherapy.	
Profiling of 742 miRNAs in plasma-derived MVs from 78 PC patients (55 LPC, 16 mPC) and 28 HI using Exiqon miRNA qPCR panel. Validation of miR-375 and miR-141 in MV and exosome fractions from sera of 47 mPC and 72 non-recurrent PC patients by TaqMan qRT-PCR. Normalised to spike-ins.	27 differentially expressed miRNAs	**miR-107**, miR-130b, **miR-141**, miR-181a-2*, miR-2110, miR-301a, miR-326, miR-331-3p, miR-432, **miR-574-3p**, miRr-625* (PC vs HI).	Bryant et al. 2012 [106]
		**miR-107**, **miR-141**, miR-181a-2*, miR-2110, miR-301a, miR-326, miR-432, **miR-574-3p**, miR-625* (LPC vs HI).	
		miR-17*, miR-20a*, miR-23a*, miR-130b, miR-198, **miR-200b**, **miR-375**, miR-379, miR-513a-5p, miR-572, miR-577, miR-582-3p, miR-609, miR-619, miR-624*, miR-1236 (mPC vs LPC)	
Discovery: profiling of 754 miRNAs in plasma from 25 PC and 17 BPH patients by Illumina miRNA expression platform. Validation: qRT-PCR analysis of 8 selected miRNAs in plasma from 80 PC, 44 BPH and 54 HI. Normalised to U6.	let-7c, let-7e, **miR-30c**, miR-622, miR-1285	5 miRNA model: AUC = 0.860, Sn = 74.1 %, Sp = 83.8 % (PC vs HI)	Chen et al. 2012 [96]
		AUC = 0.924 (PC vs BPH)	
Profiling of 365 miRNAs in serum from 25 mCRPC patients (pooled) and 25 HI (pooled) by TaqMan Low-Density Array. Normalised to spike-ins. Additional testing of individual miRNAs by TaqMan qRT-PCR.	**miR-141**, **miR-200a**, **miR-200c**, **miR-210**, **miR-375**	Increased miRNA levels in serum samples from mCRPC patients	Cheng et al. 2013 [103]
		miR-210: correlation with PSA response to treatment	
Profiling of 699 miRNAs in serum samples from 28 patients of low-risk LPC and 26 of mCRPC by TaqMan microRNA arrays.	**miR-141, miR-375**, miR-378*, miR-409-3p	Increased levels of miR-375, miR-378* and miR-141; decreased level of miR-409-3p (mCRPC vs LPC)	Nguyen et al. 2013 [117]
miRNA profiling in the serum of 8 patients with rapid BCR and 8 patients without BCR following RP. Validation: Testing of four candidate miRNAs in 70 independent Gleason 7 PC patient serum samples, 31 of whom had relapse after RP, by qRT-PCR.	**miR-141,** miR-146b-3p, miR-194	Increased miRNA levels in serum samples from patients who had experienced BCR.	Selth et al. 2013 [99]
		miR-146b-3p (HR = 2.13) and miR-194 (HR = 2.13) were also associated with disease progression in the validation cohort.	
qRT-PCR analysis of 4 miRNAs (previously found deregulated in PC tissues) in urine samples from 36 PC patients (GS6 and GS7) and 12 HI. Normalised to RNU48.	miR-205, miR-214	Decreased miRNA levels in PC. miR-205: AUC = 0.708, miR-214: AUC = 0.743	Srivastava et al. 2013 [15]
		2 miRNA model: Sn = 89 %, Sp = 80 %	
Profiling of 742 miRNAs in plasma samples from 25 LPC (pooled) and 25 mCRPC (pooled) patients by Exiqon miRNA qPCR panel. Validation: Analysis of 10 selected candidate miRNAs in 50 individual plasma samples by qRT-PCR.	**miR-16**, **miR-141**, miR-151-3p	67 differentially expressed miRNAs.	Watahiki et al. 2013 [98]
		3 miRNA model : AUC = 0.944, Sn = 84 %, Sp = 96 %	
Profiling of 732 miRNAs in serum samples from 13 BPH and 31 PC (11 LPC + 9 N1/M1 + 11 CRPC) patients by Exiqon microRNA PCR panel I + II, V2.M.	19 differentially expressed miRNAs	miR-562/**miR-210**/miR-501-3p/**miR-375**/miR-551b model: AUC = 0.919, Sn = 84 %, Sp = 100 % (BPH vs PC)	Haldrup et al. 2014 [97]
		**miR-375**/miR-708/miR-1203/**miR-200a** model: AUC = 0.875, Sn = 75 %, Sp = 100 % (LPC vs disseminated PC)	
		let-7a/miR-210/miR-562/miR-616/miR-297 model: AUC = 0.900, Sn = 80 %, Sp = 100 % (BPH vs disseminated PC)	
miRNA profiling on docetaxel-resistant and sensitive cell lines to identify candidate circulating miRNA biomarkers and subsequent qRT-PCR analysis of 46 candidate miRNAs in plasma/serum samples collected from 97 CRPC patients before and after docetaxel treatment.	miR-20a**,** miR-20b**, miR-21,** miR-25, miR-132, **miR-146a**, **miR-200a**, **miR-200b**, **miR-200c**, miR-201b, miR-222, **miR-375,** miR-429, miR-590-5p	miR-200c/miR-200b/miR-146a/miR-222/miR-201b/miR-20a model for prediction of chemoresponse: AUC = 0.730	Lin et al. 2014 [102]
		Pre-docetaxel levels of miR-200b, miR-429, miR-200a, miR-21, miR-200c, miR-590-5p, miR-375, miR-132, miR-20a and post-docetaxel decrease/no-change of miR-20a, miR-222, miR-20b, miR-132 and miR-25 associated with poor overall survival.	
miRNA profiling in PSS from 4 BPH patients and 4 PC patients by Agilent miRNA Microarray.	miR-133b, miR- 203, **miR-221**, miR-361-3p	4 miRNA model: AUC = 0.950	Guzel et al. 2015 [14]
Validation: Analysis of 4 candidate miRNA in PSS from 23 PC and 25 BPH patients by qRT-PCR.			
Deep sequencing of plasma exosomal RNA in 23 CRPC patients and correlation with OS. Validation: qRT-PCR analysis of candidate miRNAs in a follow-up cohort of 100 CRPC patients.	**miR-375**, miR-1290	miRNA levels significantly associated with poor overall survival.	Huang et al. 2015 [107]
		Combination of ADT failure time and PSA level at time of CRPC stage with miRNA levels improved predictive performance with AUC increase from 0.660 to 0.730.	
qRT-PCR analysis of 12 miRNAs in blood and tissue samples from 75 PC and 27 BPH patients	**let-7a, miR-141**, miR-145, miR-155	4 miRNA model: AUC = 0.783, Sn = 97 %, PPV = 80 %	Kelly et al. 2015 [123]
qRT-PCR analysis of 3 candidate miRNAs in cell-free urine fraction from 71 PC patients and 18 HI.	miR-483-5p	Elevated miRNA levels in PC patients, AUC = 0.694	Korzeniewski et al. 2015 [57]
Discovery: miRNA profiling in cell-free urine from 14 PC and 5 BPH patients. Validation: qRT-PCR analysis of candidate miRNAs in urine in 3 validation cohorts including 593 PC patients and 459 controls.	Hsv1-miR-H18, hsv2-miR-H9-5p	Hsv1-miR-H18: AUC = 0.772, Sn = 66.5 %, Sp = 74.1 %	Yun et al. 2015 [104]
		Hsv2-miR-H9-5p: AUC = 0.777, Sn = 70.2 %, Sp = 72.0 %	
qRT-PCR analysis of 21 miRNA in serum from 50 low-grade (Gleason grade 3) PC and 50 high grade (Gleason grade 4 + 5) PC.	**let-7a, miR-24, miR-26b**, **miR-30c**, **miR-93**, miR-100, **miR-103**, **miR-106a**, **miR-107**, miR-130b, **miR-146a**, **miR-223**, **miR-451**, **miR-874**	Highly expressed in BPH and low-grade PC, uniformly low levels in high-grade PC. 11 miR model: NPV of 0.939 for prediction of absence of high-grade PC.	Mihelich et al. 2015 [101]

*ADPC* androgen-dependent prostate cancer*, BCR* biochemical recurrence*, BPH* benign prostatic hyperplasia, *CRPC* castration resistant prostate cancer*, GS* Gleason score*, HI* healthy individuals, *mPC* metastatic prostate cancer, *LPC* localised prostate cancer, *MV* microvesicle*,* *PC*, prostate cancer, *PSS* prostate secretion samples, *qRT-PCR* quantitative reverse transcription-polymerase chain reaction, *RP* radical prostatectomy, *Sn* sensitivity, *Sp* specificity. miRNAs identified in more than one study are marked in bold

Several groups have performed miRNA profiling in plasma or serum of patients with localised or metastatic prostate cancer, BPH and healthy individuals resulting in the identification of miRNA signatures with remarkably high diagnostic value. For example, Chen et al. performed miRNA profiling in plasma from patients with prostate cancer or BPH using Illumina’s miRNA microarray and identified a 5 miRNA-model that could differentiate prostate cancer from BPH with AUC of 0.924 and prostate cancer from healthy individuals with AUC of 0.860 in an independent validation cohort. These miRNAs were shown to improve the diagnostic performance of the PSA test [96]. Similarly, by profiling miRNAs in serum, Haldrup et al. identified another 5 miRNA-panel that discriminated between prostate cancer and BPH with AUC of 0.919 [97]. Such miRNAs could potentially aid in early detection of localised prostate cancer, however whether or not they can differentiate clinically significant from indolent cancers remains to be determined.

A number of studies have identified cell-free miRNAs that differentiate between localised and metastatic prostate cancer or correlate with the risk score or Gleason grade. Such miRNAs are potentially associated with aggressive or indolent disease and may aid in tumour staging and treatment decisions at the time of diagnosis. For example, a 3 miRNA model comprising miR-141, miR-151-3p and miR-16 could differentiate localised prostate cancer from mCRPC with AUC of 0.944 [98]. Another study showed that high levels of miR-146b-3p and miR-194 in serum could predict rapid biochemical recurrence after radical prostatectomy in a cohort of 70 patients of intermediate risk according to D’Amico risk stratification system. Hence these miRNAs could help in the treatment decisions for intermediate risk localised prostate cancers [99]. Three other cell-free miRNAs, miR-106a, miR-93 and miR-1274a were found to be steadily increased, while miR-24 was steadily decreased in sera from healthy controls compared to patients with low and intermediate risk to metastatic disease [100]. Another set of 14 miRNAs was found to be highly expressed in sera of patients with BPH and low-grade (100 % Gleason grade 3) prostate cancer while had uniformly low levels in patients with high-grade cancers (Gleason grade 4 and 5) and thus could predict absence of high-grade cancer with negative predictive value of 0.939 [101]. It has to be noted that there are substantial discrepancies between studies reporting miRNAs with prognostic significance. For example, miR-106a, miR-93 and miR-451 have been shown to be highly expressed in BPH and low-grade cancers compared to high-grade cancers in one study [101], while the same miRNAs were shown to be elevated in high-risk cancers as compared to low-risk cancers and healthy controls in another study [100]. Whether such differences are due to the different risk stratification systems or technical variations in miRNA analysis is not clear at the moment.

A few studies have reported an association of cell-free miRNA levels with response to therapy. It has been shown that CRPC patients non-responding to docetaxel chemotherapy had higher levels of miR-200 family members and lower levels of miR-17 family members in plasma and serum prior to docetaxel therapy, and identified a 6 miRNA model that could distinguish responders from non-responders with AUC of 0.730 [102]. Another study showed that lower serum miR-210 level in mCRPC patients correlated with PSA response to ADT combined with chemotherapy and suggested that increased miR-210 level may serve as a marker for hypoxia response in the tumour [103].

More recently, several studies have explored the possibility of using other biofluids such as urine or prostatic secretions as a source of cell-free miRNAs. Guzel et al. were the first to demonstrate that diagnostically relevant miRNAs are present in prostate secretions. Three miRNAs were significantly downregulated and 1 was upregulated in prostate secretion samples of prostate cancer patients compared to BPH, and the combination had an AUC of 0.950 [14]. This study, was based on a small sample size and has to be validated in a larger independent cohort. Urine is an easily accessible sample type that typically is available in large amounts. Three studies demonstrated that cell-free miRNAs can be readily detectable in urine and revealed several miRNAs with a diagnostic significance [15, 57, 104]. Interestingly, the urinary virus-encoded miRNAs hsv1-miR-H18 and hsv2-miR-H9-5p could distinguish prostate cancer from BPH better than the PSA test in patients in the PSA gray zone and may aid in early detection of localised cancers [104]. However, urine is just emerging as a novel source of miRNA biomarkers and currently a direct comparison of the cell-free miRNA repertoire in blood and urine of prostate cancer patients is not available. It is too early to conclude which sample type is more suitable for the detection of miRNA biomarkers.

Most of the studies here presented used total RNA extracted from whole plasma, serum or other biofluids, while a few studies focused on EVs. Analysis of the miRNA profile in exosomes released by prostate cancer cells revealed a high degree of similarity between the miRNA of exosomes and parent cells, while a small fraction of miRNAs appeared to be specifically sorted or excluded from exosomes [105]. Bryant et al. performed miRNA profiling in MV-enriched EV fractions isolated from plasma or serum of prostate cancer patients and controls and identified miRNA panels that were differentially expressed between prostate cancer patients and healthy controls or between patients with localised and metastatic cancer [106]. These panels included some miRNAs (such as miR-141, miR-107, miR200b and miR-375) that had previously been found in studies of whole plasma or serum, yet the majority of the miRNAs did not overlap with other studies. Interestingly, miR-141 and miR-375 had similar expression patterns both in MV and exosome-enriched EV fractions [106]. Huang et al. performed deep sequencing of exosomal RNAs in CRPC patients and identified two miRNAs – miR-375 and miR-1290 that were significantly associated with overall survival and thus may aid in the treatment decisions for CRPC patients [107]. These studies show that miRNA analysis in various EV fractions isolated from blood is feasible, however, whether or not EV-based analysis provides any advantages over whole plasma or serum analysis is not yet clear.

### Technical challenges in testing cell-free miRNAs

Some cell-free miRNAs, including miR-141, miR-375, miR-21, miR-107 and miR-221, have been identified in multiple studies that strongly support their relevance as prostate cancer biomarkers. However, more than a half of the miRNAs have been associated with prostate cancer diagnosis or prognosis only in 1 study and others have been reported to have opposite prognostic roles. This could be attributed to some extent to variations in pre-analytical and analytical techniques for miRNA analysis.

Although initial studies suggested that serum and plasma miRNAs remain stable and protected from degradation after treatment with exogenous RNase A, several freeze-thaw cycles and extreme pH conditions [11, 12], later studies show that blood processing conditions may substantially influence cell-free miRNA levels [108]. A major factor affecting miRNA abundance appears to be a residual platelet contamination. It has been shown to affect the levels of 72 % of circulating miRNAs, and some of them exhibited even 1000-fold variation solely due to differences in processing [108]. The platelet count is likely to be affected by centrifugation conditions, variations in blood collection procedure and storage conditions. Furthermore, thrombocytosis is commonly found in cancer patients and has been associated with poor prognosis in various cancers [109, 110], and thus may cause a systematic bias in case-control studies [108]. In addition, levels of some miRNAs have also been shown to be affected by haemolysis [111]. This emphasizes the importance of rigorously standardised procedures for blood collection and processing, as well as controlling for haemolysis and platelet counts in studies investigating cell-free miRNAs. Factors affecting miRNA abundance and stability in other biofluids should also be systematically studied.

The choice of reference genes and/or normalisation method for qRT-PCR can also cause a systematic bias and inconsistency in the quantification of cell-free miRNAs. Compared to miRNA expression analysis in tissues, where the selection of internal controls for data normalisation is relatively straight-forward and panels of reliable controls have been established, there is no consensus on the most appropriate normalisation method for the quantification of cell-free miRNAs in biofluids. The most commonly used internal controls such as rRNAs, snoRNA, RNU6B and miR-16 have been shown to be highly variable in biofluids [112–114]. Therefore, many studies are using “spike-ins” – synthetic RNAs with no sequence homology to human miRNAs that are spiked into the biofluid sample prior to RNA extraction and amplified together with the target miRNAs. Spike-ins can control for variations arising during RNA extraction, reverse transcription and PCR efficiency, but cannot detect variations caused by platelet contamination or haemolysis. Several recent studies have made attempts to identify reliable internal controls in various biofluids by analysing large-scale expression datasets. For example, Schlosser et al. performed global profiling of miRNAs in plasma from pulmonary hypertension patients and healthy subjects and identified miR-142-3p and miR-320a as the most suitable internal controls, however it remains to be determined if these miRNAs are suitable controls in other diseases [114]. Huang et al. analysed RNA sequencing data from plasma exosomal RNAs in 192 subjects and found miR-30a-5p and miR-30e-5p as the best endogenous controls for data normalisation [107]. Finding a suitable internal control for urinary miRNA analysis seems to be even more challenging. Given that urine samples vary greatly in their concentration and volume, spike-ins are unlikely to be a suitable approach. Some of the commonly used controls such as miR-16, RUN6-2, miR-518a and miR-3605 exhibited large variation between urine samples and therefore are not suitable as urinary reference genes. Thus, at the moment, normalisation to the total RNA concentration seems to be the most reliable approach [104].

## Conclusions

Eight years ago cell-free miRNAs emerged as an entirely new type of cancer biomarkers detectable in human biofluids. Since then, a number of cell-free miRNAs that may serve as biomarkers of prostate cancer has been discovered. Most of them are putative diagnostic or prognostic biomarkers that may aid in early detection or help to distinguish aggressive cancers from indolent cancers. To date, far less predictive biomarker candidates that may aid in therapeutic choices for advanced cancers have been discovered.

The sample sizes are relatively small in most of the studies and the identified miRNA biomarkers should be validated in cohorts with adequate statistical power and in a clinically relevant setting. Biomarkers that are expected to detect early-stage cancers or be associated with aggressiveness should be evaluated in longitudinal studies to assess at what time point during disease development a candidate biomarker becomes detectable in biofluids. This would show if a putative prognostic biomarker appears in the biofluid only when the cancer already has metastasized or before the clinically detectable metastases appear, and therefore could predict the disease behaviour. Next, the performance of a biomarker assay should be evaluated in a blinded, randomised clinical trial, before it can be used in a clinical setting.

Most studies have explored cell-free miRNAs in blood. However, several recent studies demonstrated that cancer-associated cell-free miRNAs can also be detected in other biofluids, such as urine or prostate secretions, which potentially may be enriched in cancer-derived miRNAs and have a lower background of miRNAs released by various normal cells. To evaluate which biofluid is the best source of prostate cancer-associated miRNAs, a systematic comparison of miRNA profiles in blood, urine, prostate secretions and cancer tissues of the same patient is required.

It has been suggested that purified EVs may have several advantages over whole-plasma (or other biofluid) analysis since they may contain cancer-associated miRNA signatures and provide better protection against degradation. In addition, prostate cancer-derived exosomes have been shown to be enriched in PSMA [115], a prostate-specific membrane antigen that is upregulated in a vast majority of prostate cancers [116]. Hence, PSMA might serve as a tool for detection and isolation of prostate cancer-derived exosomes from biofluids. This, in turn, could enable the analysis of cancer-derived miRNAs and other nucleic acids without contamination of those derived from normal cells. Nevertheless, it is still an open question what type of EVs represents the best source of miRNA biomarkers and whether EV isolation can improve the detection of prostate cancer-associated miRNAs in biofluids. A head-to-head comparison of EV-based versus whole biofluid-based techniques would be highly relevant to address this question.

The cellular origin of cell-free miRNAs is also an important aspect. Initial studies suggested that the majority of cell-free miRNAs in the blood of healthy individuals is released from blood cells, while disease-associated miRNA signatures may be derived from the tissues affected by the disease [11, 12]. Several miRNAs, such as miR-141, miR-375, miR-200a, miR-200c and miR-210, which were found at elevated levels in blood of prostate cancer patients, have also been shown to be overexpressed in prostate cancer tissues [103, 117], suggesting that these circulating miRNAs originate from prostate cancer tissues. However, no such correlation has been found for other miRNAs, such as miR-378* and miR-409-3p [117]. The cellular origin of miRNAs that are decreased in biofluids is even more controversial as the decrease is very unlikely to be related to their expression level in tumour tissues. Instead, it might be associated with inflammatory or immune responses to the tumour.

Taking together, these studies suggest that cell-free miRNAs are a novel and very attractive type of cancer biomarkers. Gaining a deeper understanding of the questions arising from the initial studies will help to design future miRNA biomarker discovery studies, assess the identified biomarker candidates and select the best candidates for evaluation in clinical trials.

## Abbreviations

ADT: androgen deprivation therapy
AUC: area under the curve
BPH: benign prostatic hyperplasia
EV: extracellular vesicle
mCRPC: metastatic castration-resistant prostate cancer
miRNA: microRNA
MV: microvesicle
PSA: prostate specific antigen
PSMA: prostate-specific membrane antigen
